# The International Conference on Intelligent Biology and Medicine 2018: Medical Informatics Thematic Track (MedicalInfo2018)

**DOI:** 10.1186/s12911-019-0732-0

**Published:** 2019-01-31

**Authors:** Yaoyun Zhang, Cui Tao, Yang Gong, Kai Wang, Zhongming Zhao

**Affiliations:** 1 Center for Computational Biomedicine, School of Biomedical Informatics, The University of Texas Health Science Center at Houston, Houston, TX 77030 USA; 2 School of Biomedical Informatics, The University of Texas Health Science Center at Houston, Houston, TX 77030 USA; 3 Raymond G. Perelman Center for Cellular and Molecular Therapeutics, Children’s Hospital of Philadelphia, Philadelphia, PA 19104 USA; 4 Department of Pathology and Laboratory Medicine, University of Pennsylvania, Philadelphia, PA 19104 USA; 5 Center for Precision Health, School of Biomedical Informatics, The University of Texas Health Science Center at Houston, Houston, TX 77030 USA

## Abstract

In this editorial, we first summarize the 2018 International Conference on Intelligent Biology and Medicine (ICIBM 2018) that was held on June 10–12, 2018 in Los Angeles, California, USA, and then briefly introduce the six research articles included in this supplement issue. At ICIBM 2018, a special theme of Medical Informatics was dedicated to recent advances of data science in the medical domain. After peer review, six articles were selected in this thematic issue, covering topics such as clinical predictive modeling, clinical natural language processing (NLP), electroencephalogram (EEG) network analysis, and text mining in biomedical literature.

## Introduction

The 2018 International Conference on Intelligent Biology and Medicine (ICIBM 2018) was held on June 10–12, 2018 in Los Angeles, California USA. ICIBM 2018 included four keynote lectures, four eminent scholar talks, four tutorials, eleven regular scientific sessions, and one poster session. The scientific sessions, papers and abstracts cover a great variety of research topics in bioinformatics, systems biology, intelligent computing, data science, and medical informatics.

Nowadays, the rapidly growing Electronic Health Records (EHRs), biomedical literature and other healthcare data have generated large amount of information to facilitate significant discoveries in clinical and translational research. At ICIBM 2018, a special theme of “Medical Informatics” was dedicated to recent advances of data science in the medical domain. It was especially attractive to the conference participants, both from paper and abstract submission and the onsite discussion during the conference session. Here, we present a summary of the 6 research articles that were selected for the ICIBM 2018 supplement session on Medical Informatics. All the papers underwent peer-review and revision before final acceptance.

## Summary of selected papers in the thematic issue

The six papers selected for this thematic issue were presented at the ICIBM 2018. These papers cover a wide range of topics including clinical predictive modeling, clinical natural language processing (NLP), electroencephalogram (EEG) network analysis, and mining of gene and disease relation in biomedical literature.

Clinical predictive modeling based on different types of healthcare data has a wide range of applications, including medical resource optimization, decision making and healthcare quality measurement, among others. Zimmerman et al. [1] developed and validated a data driven multivariable clinical predictive model for early detection of acute kidney injury (AKI) among a large cohort of adult critical care patients. Using demographic, clinical data, and laboratory test measurements from Day 1 of ICU (Intensive Care Unit) admission, the model can accurately predict max serum creatinine level during Day 2 and Day 3, which has the potential to assist clinicians in identifying patients at greater risk of new onset of AKI in critical care setting. Yang et al. [2] designed a dynamic random survival forest model to continuously predict the survival risk of hospitalization caused by congestive heart failure using periodically updated claim data. The method has achieved a better accuracy than several related and popular methods; thus, it may help prevent possible hospitalization in the future.

Relation recognition between clinical concepts in text is one important NLP task for clinical applications, which is challenging and has limited performance for practical use. To address this problem, various deep learning (neural network) architectures are proposed to leverage syntactic information for relation extraction. As the first study on Chinese clinical notes, Liu et al. [3] designed a recurrent convolutional neural network (RNN-CNN) method to recognize the temporal relations between medical entities and temporal expression. Li et al. [4] proposed a novel neural approach to model shortest dependency path between target entities together with the sentence sequence for clinical relation extraction. Experimental results show that the proposed approach achieved significant improvements over comparable existing methods, demonstrating the effectiveness of utilizing syntactic structures in deep learning-based relation extraction.

The construction of dynamic EEG network diagram plays an important role in the visualization of brain discharge. Mei et al. [5] provided a feasible construction method by mapping the synchronization of the nonlinear characteristics of EEG. By analyzing the network core nodes of different seizure states in patients with epilepsy, the results indicated that the path of EEG synchronous propagation was closely related to the localization of the epileptic foci. This study could help to develop the epileptic network and may contribute to the understanding of information dissemination mechanism of the cerebral neural network.

Revealing the associations between diseases and genes as well as between diseases may help to understand the mechanisms of diseases, improve the prevention and treatment of diseases, and support the discovery of drug repurposing. Chen et al. [6] introduced a mathematic model to quantitatively measure the associations between genes and diseases, which was then used to indicate the relations between diseases. By applying this approach to analyze the relationships between Chronic Obstructive Pulmonary Disease (COPD) and other diseases under the Lung diseases branch in MeSH (the Medical subject heading index system), 4 novel diseases relevant to COPD were detected. As judged by domain experts, the F-score of the approach is up to 77.6%, indicating a very good performance.

## Discussion and conclusion

Medical informatics, or health informatics, is the study of using data and information technology to deliver health care services of better qualities. Over the past decade, large amounts of healthcare data, especially the detailed longitudinal patient information, have been accumulated and are available electronically. Tremendous efforts and resources have been devoted to develop infrastructures for secondary use of healthcare data and have resulted in significant discoveries in clinical and translational research.

The goal of this workshop is to bring experts in the field of health informatics to discuss innovative methods, applications, and tools to facilitate medical and translational research and address important healthcare problems. As shown in this workshop, diverse types of data including EHRs, claim data, EEG data and biomedical literature have been studied. Moreover, new methods and algorithms (e.g., various deep learning algorithms) have been developed to process, integrate, and analyze these data into knowledge and actionable wisdom, thus to facilitate medical research and to improve clinical practice. We envision a rapid growth of the medical informatics field and hope to organize more such events to promote medical informatics research in the big data era.
